# Effect of a probiotic containing lactobacilli and bifidobacteria on the metabolic processes, litter microbiocenosis, and production indicators of broiler Pekin ducklings

**DOI:** 10.14202/vetworld.2022.998-1005

**Published:** 2022-04-20

**Authors:** Ayrat Khabirov, Ruzil Avzalov, Gulnara Tsapalova, Alexandra Andreeva, Almaz Basharov

**Affiliations:** Department of Physiology, Biochemistry and Feeding Animals, Federal State Budgetary Educational Establishment of Higher Education “Bashkir State Agrarian University,” Ufa, Russian Federation

**Keywords:** metabolism, microbiocenosis, Pekin ducks, probiotic supplementation, production

## Abstract

**Background and Aim:**

The use of antibiotics is prohibited in poultry feeding in many countries worldwide, which has resulted in the emergence of antibiotic-resistant bacteria. Therefore, probiotic supplements are a good alternative in these circumstances. Probiotics, including *Bifidobacterium adolescentis* and *Lactobacillus acidophilus*, can reduce the development of resistance and stimulate the growth of broiler ducklings. This study aimed to evaluate properties of the probiotic supplement containing these bacteria.

**Materials and Methods:**

We studied the response of broiler ducklings to the addition of a dietary probiotic supplement containing lactobacilli and bifidobacteria until they reached 7 weeks of age. All birds participating in the experiment received probiotic supplements on days 1-7, 15-21, 29-35. The state of the ducklings was assessed on day 21. At the age of 21 and 42 days, 4-5 ml of blood was drawn from the wing vein of 10 randomly selected birds (5 in each group). Blood samples were analyzed for total protein, concentration of glucose, hemoglobin, calcium and inorganic phosphorus, as well as the number of erythrocytes and leukocytes. Enzymatic calorimetric method, molybdate method, and haemocytometry according to Nutt method and haemoglobin cyanide method were used to analyze blood samples.

**Results:**

The live weight of the experimental ducklings increased by 5.0%, showing a positive effect of probiotic supplementation, whereas their feed consumption per kilogram of weight gain decreased. Their hemoglobin content and red blood cell count increased. Although the number of lactobacilli and bifidobacteria increased, the number of *Escherichia coli* cells decreased 2.15-fold (p<0.05). The addition of the probiotic supplement contributed to improving the digestibility of protein and fat by 1.6%, fiber by 3.4%, and nitrogen-free extractive substances by 4.7%. The broiler ducklings had high meat quality indicators, including dressing percentage, which increased by 5.4%, whereas the fat content decreased by 1.3%.

**Conclusion:**

The introduction of the probiotic supplement in the diet of broiler ducklings improved their growth indicators and increased the number of lactobacilli and bifidobacteria while decreasing the number of *E. coli* cells in the intestine. It not only improved the meat quality but also increased the profit from $0.392 per bird in the control group to $0.472 per bird in the experimental group. Therefore, this probiotic supplement is a good alternative for raising ducklings in large enterprises and farms. The study limitations may be that the results are only applicable to broiler ducklings. The use of lactobacilli may yield different results in other bird breeds or broiler ducklings in different age groups.

## Introduction

One of the critical problems of modern society is providing food to people. A comprehensive solution to complex agro-industrial problems, particularly animal husbandry and poultry farming, is the effective use of production resources [1,2]. Food products of animal origin, including meat from farm animals and poultry, play an essential role in human diet. Thus, farmers should aim to increase meat production in large-scale livestock farms. Although the practice of using feed antibiotics remains common, some countries have prohibited their use. As such, producers are searching for alternatives to ensure that meat products are environment-friendly and of high quality.

An urgent problem regarding the use of antibiotics is the emergence of antibiotic-resistant bacterial strains, which increase the incidence of animals with dysbiosis; therefore, with a decline in the efficacy of antibiotics, it has become challenging to treat these animals [3-6]. Among antibiotic alternatives, the use of live microorganisms to stabilize the microflora of the gastrointestinal tract is now common; they are added to mono- or multicomponent probiotic supplements immobilized on various sorbents [7].

Novel feed additives, such as probiotics and prebiotics, help regulate intestinal microbiota [3]. The use of a synbiotic containing raw materials of medicinal plants and lactobacilli in newborn calves and piglets during weaning was shown to increase the activity of bifidoflora and lactoflora and suppress the growth of *Escherichia coli*, enterococci, staphylococci, and clostridium [4]. Livestock productivity and feed efficiency are closely associated with qualitative and quantitative microbial burden on animal intestines, the morphological structure of the intestinal wall, and immune system functioning, which can be adjusted through the use of probiotics and prebiotics [7]. Feed additives containing probiotics positively affect the amino acid and mineral composition of broiler meat [8]. Furthermore, diets containing *Bacillus subtilis* improve the growth rates, serum immunoglobulin level, intestinal microbiocenosis, and resistance to *E. coli* in chickens [9]. A study also investigated the effects of probiotic supplements containing different dietary doses of *Bacillus* strains on the growth and feed efficiency of broiler chickens [10]. The effectiveness of probiotic supplements included in animal diets has also been studied [11,12].

The normal microflora of the gastrointestinal tract plays a crucial role in protecting against pathogenic microorganisms by stimulating the immune system and participating in metabolic reactions. The gastrointestinal tract is a competitive environment for pathogenic microflora; the latter is suppressed through binding to the surface receptors of cells, especially epithelial cells. These antagonistic qualities are most pronounced in lactobacilli and bifidobacteria. Their antibacterial potential is realized as a result of the secretion of lysozyme, alcohols, bacteriocins, and other compounds. High concentrations of lactobacilli and bifidobacteria weaken the metabolism and release of toxins by pathogenic bacteria.

In the gastrointestinal tract, bifidobacteria suppress the development of putrefactive microorganisms and positively affect carbohydrate metabolism. Bifidobacteria, which adhere to the intestinal mucosa, create a protective barrier for the infiltration of pathogenic microflora into the body. Because of the production of organic fatty acids, bifidobacteria suppress the activity of pathogenic microflora; synthesize vitamins, amino acids, and proteins; and participate in the absorption of iron and calcium (Ca) ions through the intestinal wall.

Lactobacilli are the main representatives of intestinal microbiocenosis. They ensure the conversion of lactose into lactic acid, which prevents the growth of pathogenic bacteria. Lactobacilli have an antagonistic effect on staphylococci, *E. coli*, and proteus. Lactobacilli affect the body’s metabolic processes, increasing their non-specific resistance. Because of the role that lactobacilli play in metabolic processes, studying these processes and their manifestations (e.g., changes in body weight) is important.

This study aimed to investigate the response of broiler ducklings to a probiotic supplement containing lactobacilli and bifidobacteria. The objectives of this study were to investigate and assess the live weight gain, hematological and biochemical blood serum parameters, feed intake and nutrient digestibility, microbiocenosis changes, and meat quality. An economic analysis of the results was also conducted.

## Materials and Methods

### Ethical approval

The committee for animal care and use of the Federal State Budgetary Educational Establishment of Higher Education “Bashkir State Agrarian University” approved all procedures for the use of animals in this study.

### Study period and area

This study was conducted from March 2019 to September 2019 in the “Blagovarsky Plemptitsezavod” production conditions of the Republic of Bashkortostan, Russia.

### Animals

Pekin crossbreed “Agidel” ducklings were used in this study. These ducklings were bred in incubators of this enterprise, which is a breeding and genetic center for water birds. One-day-old ducklings were randomly selected under production conditions. The ducklings were categorized into two groups: (1) control group, which included 40 ducklings; and (2) experimental group, which included 40 ducklings who received a probiotic supplement containing lactobacilli and bifidobacteria on the following days of feeding: from the 1^st^ to 7^th^, 15^th^ to 21^st^, and 29^th^ to 35^th^ days.

During the 1^st^ week of the ducklings’ life, the temperature in the poultry house was 26°C–28°C and the relative humidity was 65%–70%. In the 2^nd^ week, the temperature was decreased by 2°C–3°C. By the end of the 3^rd^ week of life, the temperature was maintained at 22°C. Subsequently, the temperature was maintained at 18°C–20°C. The ducklings were raised on the floor, with sawdust used as bedding material. For the first 7 days, the poultry houses were kept lit day and night. When the ducklings were 6 weeks old, the lighting period was reduced to 8 h/day.

### Feed characteristics

The ducklings received a full-fledged compound feed. The feed recipe from days 1 to 21 (per 100 g of feed) consisted of the following: crushed wheat, 62 g; sunflower meal, 12 g; grain mixture, 25 g; lime phosphate, 1 g; vitamin premix, and trace elements. The feed recipe from days 22 to 31 (per 100 g of feed) consisted of the following: crushed wheat, 68 g; sunflower meal, 12 g; grain mixture, 25 g; lime phosphate, 2 g; vitamin premix, and trace elements. The feed recipe from days 32 to 42 (per 100 g of feed) consisted of the following: crushed wheat, 71 g; sunflower meal, 12 g; grain mixture, 15 g; lime phosphate, 2 g; vitamin premix, and trace elements.

The feed for 1-day to 3-week-old ducklings consisted of crushed wheat (62%), sunflower meal (12%), grain mixture (25%), tricalcium phosphate (1%), vitamin premix, and trace nutrients. The quality indicators were exchange energy (EE), 280 kcal; crude protein (CP), 23.0%; Ca, 1.2%; and phosphorus (P), 0.8%. From days 22 to 31, the quality indicators were EE, 280 kcal; CP, 20.0%; Ca, 1.2%; and P, 0.8%. From days 32 to 42, the quality indicators were EE, 280 kcal; CP, 19.0%; Ca, 1.2%; and P, 0.8%. During the first week, the ducklings were fed every 2 h. All the ducklings had free access to a drinking pan.

### Probiotic based on lactobacilli and bifidobacteria

The probiotic supplement contained *Bifidobacterium adolescentis* (at least 80 million/g) (Component, Russia) and *Lactobacillus acidophilus* (at least 1 million/g) (Component). Other components of the probiotic supplements were amino acids, vitamins, organic acids, and trace nutrients. The probiotic supplement was a light brown powder and was introduced into the compound feed during the stepwise premixing process.

### Blood biochemical parameter analysis

At 21 and 42 days of age, 4–5 mL of blood was taken from the wing vein of each bird blood samples of 10 randomly selected ducklings (five ducklings from each group).

The experimental ducklings received a probiotic supplement periodically on the following days: days 1–7, 15–21, and 29–35. The condition of the ducklings was assessed at the end of the second period of probiotic administration (on day 21). For ducklings of this age, the lowest feed consumption per 1 kg of live weight gain and average daily growth rates were close to the maximum values for the entire growing period (maximum: 4 weeks). Blood sampling was conducted on day 42 to assess the condition of the ducklings at the end of the growing period, when they reached the slaughter weight.

Heparin was used as an anticoagulant. Plasma samples were obtained by centrifugation at 2000 rpm for 10 min. The samples were transported to a laboratory in a thermal insulation bag (24°C–26°C) within 2–2.5 h after taking and examining them on the same day. The transforming solution was composed of K_3_Fe (CN)_6_, 200 mg; KSN, 50 mg; and KH_2_PO_4_; 140 mg.

Plasma samples were analyzed using analytical kits (STAT FAX^®^ 3300, USA), according to the manufacturer’s instructions (Vital Diagnostics; Vital Development Corporation, Russia). The total protein content of the blood serum was evaluated according to the manufacturer’s instructions (Vital Diagnostics); this method is based on the formation of a colored complex with copper ions in an alkaline medium. The glucose concentration was determined using the enzymatic colorimetric method with a Glucose-VITAL diagnostic kit (Vital Diagnostics) and a biochemical analyzer (STAT FAX^®^ 3300). When β-D-glucose is oxidized by atmospheric oxygen under the action of glucose oxidase, an equimolar amount of hydrogen peroxide is formed. Hydrogen peroxide oxidizes chromogenic substrates to form a colored product.

#### Unified colorimetric method

The Ca concentration in the blood serum was determined using the unified colorimetric method with a Calcium-VITAL diagnostic kit (Vital Development Corporation) and a biochemical analyzer (STAT FAX^®^ 3300). Ca forms a colored complex with an o-cresol-phthalein complex in an alkaline medium.

#### Molybdate method

The inorganic P concentration in the blood serum was determined using the molybdenum method (ammonium molybdate is a reagent) using a Phosphorus-VITAL diagnostic kit (Vital Development Corporation) and a biochemical analyzer (STAT FAX^®^ 3300). In this method, inorganic P interacts with ammonium and forms a phosphomolybdate complex in a sulfuric acid solution.

### Analysis of blood morphological parameters

The hemoglobin concentration was determined using a unified hemoglobin cyanide method [13]. Approximately 20 mL of blood was added to 5 mL of the transforming solution, thoroughly mixed, and left for 10 min. The optical density of the experimental sample was measured using an analyzer (STAT FAX^®^ 3300).

The total number of leukocytes and erythrocytes was calculated by hemocytometry using Natt and Herrick blood cell dilution fluid [13]. Leukocytes from thin smears were counted using the Leishman staining technique in a Goryaev chamber.

### Microbiological analysis

The poultry litter was analyzed using elective and differential media. Lactobacilli were cultured in a nutrient medium using MRS-2 agar (Research Center for Pharmacotherapy, St. Petersburg, Russia) and incubated at 37°C for 48 h. Bifidobacteria were isolated from large dilutions using Blaurock nutrient medium (Research Center for Pharmacotherapy). *E. coli* was cultured on Endo agar (Research Center of Pharmacotherapy). The number of colony-forming units was expressed as a logarithm of colony-forming units per gram of intestinal contents.

### Analysis of poultry anatomical dressing

The objective of raising broiler ducklings was to obtain high-quality meat products. However, meat production indicators must be improved by using probiotic supplements to provide ecological safety in poultry farming.

On day 42, three ducklings from each group were randomly selected and slaughtered, and their carcasses were anatomically dissected as per described by Campbell *et al*. [13]. The following indicators were evaluated: (1) pre-slaughter weight, i.e., the weight of the live bird before slaughter; (2) half-cut carcass mass, i.e., bloodless carcass, without feathers, with an extirpated intestine and cloaca, without a gorge and an oviduct (in females); and (3) cut carcass mass, i.e., bloodless carcass, without feathers, internal organs, gastrointestinal tract, beheaded, and legs cut up to the ankle joint.

### Statistical analysis

Statistical analysis was performed using Statistica 10 (Statsoft Inc., USA). Quantitative data were presented as the arithmetic mean and standard error (M ± m). Differences between groups were determined using the Student’s *t*-test and were considered statistically significant at p < 0.05.

## Results

### Dynamics of duckling growth with a probiotic supplement

The dietary probiotic supplement containing lactobacilli and bifidobacteria positively affected the ducklings’ live weight gain dynamics (Table-1). During the 1^st^ week of life, there were no significant differences in the live weight gain between the control and experimental groups. At the end of the 3^rd^ week of life, the live weight of ducklings in the control group was 1077.4 ± 89.6 g, which indicates a tendency to increase this indicator in the experimental group by 2.5% (p≤0.05). However, by the end of the 4^th^ week, the ducklings in the experimental group had a relatively large weight. The average daily gain (ADG) in the experimental ducklings was 107.3 g/day and in the control ducklings was 90.3 g/day (p≤0.05). In the final week, the ADG in the experimental ducklings was 9.1% higher than that in the control group (p≤0.01), reaching 74.6 g/day. At 42 days of age, the live weight of the experimental ducklings was 2814.8 g, which exceeded the live weight of the control ducklings by 5.0% (p≤0.05).

**Table 1:** Dynamics of live weight of ducklings when using the probiotic “Lactobifadol” (X ± Sx, n=40).

Age (weeks)	Group	Live weight, g	Average daily weight gain, g
0	Control	57.3±1.23	
	Experimental	57.2±1.77	
1	Control	173.4±14.8	16.7±1.84
	Experimental	173.7±14.4	16.7±2.18
2	Control	531.5±34.6	51.1±3.45
	Experimental	480.2±52.3	43.9±3.49
3	Control	1077.4±89.6	78.0±6.74
	Experimental	1051.3±84.3	81.6±8.57
4	Control	1709.2±105.3	90.3±9.87
	Experimental	1802.6±101.8	107.3±11.65
5	Control	2202.3±108.6	70.4±6.84
	Experimental	2291.7±121.7	69.9±5.69
6	Control	2681.6±114.3	68.4±5.42
	Experimental	2814.8±127.5	74.6±5.84

**Figure 1:**
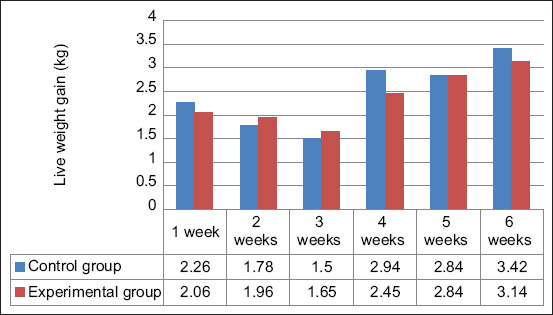
Feed consumption per 1 kg of live weight gain. The differences between the control and experimental groups are significant at p < 0.05; p<0.01, n = 40.

During the 1^st^ week of life, the feed consumption per kilogram of live weight gain in the control group was 9.7% higher than that in the experimental group (p≤0.01), with an absolute value of 2.26 kg (Figure-1). However, during the 2^nd^ and 3^rd^ weeks of life, the consumption in the experimental group was 10.2% and 10.0% higher than that in the experimental group, respectively (p≤0.01). The feed consumption in the control ducklings was higher than that in the experimental ducklings by 20.0% at week 4 (p≤0.01) and 9.0% at week 6 (p≤0.01). According to the feed conversion, the average feed consumption per 1 kg of live weight gain across 6 weeks was 4.7%, more effective in the experimental ducklings than in the control ducklings (p≤0.05).

**Figure 2:**
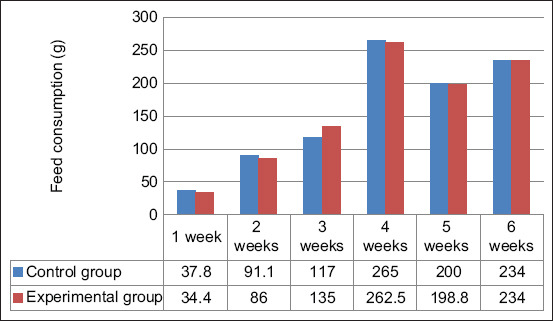
Dynamics of feed consumption per 1 head per day.

The feed consumption per head steadily increased, starting from 1 week until the end of the production process. At 4 weeks of age, the feed consumption peaked at 265 g/head/day in the control group and 262.5 g/head/day in the experimental group (Figure-2, p≥0.05). Over 6 weeks, the average consumption of compound feed per head per day did not differ between the control (157.7 g/head/day) and experimental groups (158.5 g/head/day) (p≤0.05).

### Blood morphological and biochemical parameters

Table-2 outlines the results of the blood morphological analysis of 21- and 42-day-old ducklings. At 21 days of age, the introduction of the probiotic supplement containing lactobacilli and bifidobacteria stimulated 10.7% more erythrogenesis in comparison with the control group. Moreover, in the experimental ducklings, red blood cells were higher at 42 days than at 21 days. At the same stage, the number of red blood cells was 9.5% higher in the blood obtained from experimental ducklings than in the blood obtained from control ducklings.

**Table 2:** Dynamics of the blood morphological parameters of ducklings (X ± Sx, n=5).

Indicator	Group	

	Control group	Experimental group
Age: 21 days		
Red blood cells, 10^12^/L	2.43±0.49	2.69±0.28
Hemoglobin, g/L	138.4±4.72	143.0±4.53
White blood cells, 10^9^/L	21.09±1.44	22.63±1.12
Age: 42 days		
Red blood cells, 10^12^/L	2.96±0.39	3.24±0.27
Hemoglobin, g/L	155.7±3.59	165.1±4.73
White blood cells, 10^9^/L	22.4±1.97	23.5±2.04

The hemoglobin content in the experimental ducklings (143 g/L) was 3.4% higher than that in the control ducklings (p≤0.05). This trend persisted until the ducklings were 42 days old, at which time the hemoglobin content in the experimental ducklings was 165.1 g/L, which was 6.1% higher than that in the control ducklings (p≤0.05). The absolute number of white blood cells demonstrated less variability with age; however, the white blood cell count of the experimental ducklings was 7.3% and 5.0% higher than that of the control ducklings at 21 and 42 days, respectively (p≤0.01 and p≤0.05, respectively).

The mean values of the blood morphological parameters of 21–42-day-old experimental ducklings were higher than those of the control ducklings. The number of red blood cells was higher by 10.1% (p≤0.01), white blood cells by 6.1% (p≤0.05), and hemoglobin content by 4.8% (p≤0.05). To obtain an overview of the experimental changes associated with the use of the probiotic supplement, blood serum biochemical parameters were studied; the results are presented in Table-3.

**Table 3:** Biochemical parameters of the blood serum of broiler ducklings (X ± Sx, n=5).

Indicator	Group	

	Control	Experimental
Age: 21 days		
Total protein, g/L	40.6±2.66	39.2±3.77
Albumin, g/L	22.7±2.44	23.1±2.69
Calcium, mmol/L	2.6±0.09	2.1±0.08**
Phosphorus, mmol/L	1.67±0.03	1.75±0.03*
Age: 42 days		
Total protein, g/L	55.4±4.42	56.6±4.28
Albumin, g/L	25.0±1.92	27.2±1.17
Calcium, mmol/L	2.2±0.11	2.3±0.09
Phosphorus, mmol/L	1.89±0.03	1.92±0.04

The differences between the control and experimental groups are significant at

* p<0.05;

** p<0.01

At 21 days of age, the total protein content decreased by 3.6% in the experimental ducklings (p≤0.05); however, it increased in both groups. At the same time, the total protein content in the experimental ducklings increased by 2.2% in comparison with the control ducklings (p≤0.05). The serum albumin content tended to increase with age; compared with the control ducklings. It was 1.8% and 8.8% higher in the 21- and 42-day-old experimental ducklings, respectively (p≤0.05 and p≤0.01, respectively). The serum Ca content was 23.8% higher in the control group than in the experimental group at 21 days (p≤0.001); however, at 42 days, it was 4.6% higher in the experimental group than in the control group (p≤0.05). Compared with the control ducklings, the experimental group ducklings had a 4.8% higher P content at 21 days (p≤0.05), with this difference decreasing to 1.6% at 42 days (p≤0.05).

### Litter microbiocenosis

The probiotic supplement affected the microbiocenosis of the ducklings’ litter (Table-4). The number of lactobacilli and bifidobacteria in the litter from the experimental ducklings was 1.9 times (p<0.10) and 1.57 times (p<0.10) greater than that from the control ducklings at 21 days of age. Nonetheless, the number of *E. coli* decreased 2.15 times (p<0.05).

**Table 4:** Microbiocenosis of ducklings’ litter at 21 days of age when using the probiotic supplement “Lactobifadol” l g, CFU/g (X ± Sx, n=3).

Group	Lactobacilli	Bifidobacteria	*Escherichia coli*
Control	3.6±0.85	3.7±0.62	2.8±0.34
Experimental	6.9±0.96*	5.8±0.68*	1.3±0.28**

The difference is valid at

* p<0.1;

** p≤0.05

### Nutrient digestibility

Assessments of the digestibility of feed and its nutrients are an essential aspect of poultry farming. We compared differences in the nutrient digestibility of duckling diets with and without the inclusion of the probiotic supplement; the results are shown in Table-5. Overall, the nutrient digestibility was improved with the inclusion of the probiotic supplement. The protein digestibility coefficient of the experimental ducklings increased to 68.2 compared with that of the control ducklings (66.6%; p≤0.05). The fat digestibility coefficient in the experimental group was 75.2%, which was 1.6% higher than that in the control group (73.6%; p≤0.05). The fiber digestibility was 3.4% higher in the experimental group (p≤0.05), and the digestibility of nitrogen-free extractive substances was 4.7% higher than that in the control group (p≤0.05).

**Table 5:** Coefficients of digestibility of nutrients when using the probiotic supplement “Lactobifadol” for raising ducklings at 21 days of age, % (X ± Sx, n=3).

Group	Indicator			

	Protein	Fat	Fiber	Nitrogen-free extractive substances
Control	66.6±2.04	73.6±2.86	20.3±1.39	80.6±3.14
Experimental	68.2±2.14	75.2±3.32	23.7±1.12	85.3±2.26

### Meat productivity

The results of the meat quality evaluation are shown in Table-6. The addition of the probiotic improved the meat quality of the ducklings. The semi-cut carcass yield was higher in the experimental group than in the control group (82.9% versus 79.2%; p≤0.05). Similarly, the cut carcass yield was 62.3% in the experimental group versus 56.9% in the control group and the muscle yield was 43.1% in the experimental group versus 39.6% in the control group (p≤0.05 for both). Conversely, the fat content in the experimental group was 37.4%, which was 1.3% less than that in the control group (p≤0.05).

**Table 6:** Meat productivity of broiler ducklings (n=3).

Indicator	Group	

	Control	Experimental
Pre-slaughter live weight, g	2374±45.6	2514±61.3
Weight of a semi-cut bird, g	1880±34.3	2084±64.3
Semi-cut bird yield, %	79.2±0.60	82.9±1.18
Weight of a cut bird, g	1351±24.6	1568±31.8
Cut bird yield, %	56.9±1.25	62.3±2.34
Carcass fatness, %	38.7±0.94	37.4±0.86
Total muscle yield, g	535±12.4	676±14.1***
%	39.6±0.68	43.1±0.83

The differences between the control and experimental groups are significant at

*** p<0.001, n=3

### Economic analysis

The economic efficiency of raising ducklings using a probiotic supplement can be determined based on the cost of production per unit of production (Table-7). The average slaughter weight of the control and experimental ducklings was 2681.6 g and 2814.8 g, respectively (p≤0.05). The total slaughter weight of the control and experimental ducklings was 107.264 kg and 112.592 kg, respectively (p≤0.05), indicating an increase of 5.328 kg. Expenditure accounting demonstrated that the control and experimental ducklings consumed 264.6 kg and 266.3 kg of mixed feed, respectively.

**Table 7:** Economic efficiency of the use of the probiotic “Lactobifadol” for raising ducklings.

Indicator	Group	

	Control group	Experimental group
The number of ducklings at the end of raising	40	40
Average slaughter weight of one duckling, g	2,681.6	2,814.8
± to the control group, g	-	+133.0
Total slaughter weight of ducklings, kg	107.264	112.592
± to the control, kg	-	+5.328
Feed consumption, g	264.6	266.3
Feed price (per 1 kg), RUB/(USD)	35.2/(0.470)	35.5*/(0.474)*
Total feed costs, RUB/(USD)	9313.92/(124.2)	9453.65/(126.0)
Feed cost per one bird live weight gain, RUB/(USD)	86.83/(1.16)	83.96/(1.12)
Total cost of raising broiler ducklings, RUB	13,304.3/(177.4)	13,781.3/(183.7)
Cost of 1 kg of weight gain, RUB/(USD)	124.0/(1.65)	122.4/(1.63)
Cost of selling of 1 kg of weight gain of broiler ducklings, RUB/(USD)	0.135/(1.8)	0.135/(1.8)
Sales revenues, RUB/(USD)	14,480.6/(193.07)	15,199.9/(202.67)
± to the control group, RUB/(USD)	-	+719.3/(9.59)
Profit, RUB/(USD)	1176.3/(15.68)	1418.6/(18.91)
± to the control group, %	-	20.6

* The cost of the feed includes the cost of the probiotic Lactobifadol

The cost of 1 kg of feed in the control group was 35.2 rubles (0.473 USD)/kg, whereas it was 35.5 rubles (0.474 USD)/kg in the experimental group, including the cost of the probiotic supplement. The cost of feed for 1 kg of weight gain in the control group was 86.83 rubles (1.157 USD), which was 3.5% higher than that in the experimental group. The profit gained from the sale of ducklings in the experimental group was 719.3 rubles (9.6 USD), which was 20.3% higher than that in the control group. Thus, raising ducklings using the probiotic supplement increased sales revenue by 719.3 rubles (9.590 USD), allowing for an additional profit of 20.6%.

## Discussion

Farm animal production mainly focuses on consumer needs and the production of environment-friendly and safe products. In this regard, probiotic supplement feed additives are a reasonable alternative to antibiotics. Probiotic manufacturers are working to create new formulations, some of which have proven to be effective, whereas others have not. This study aimed to evaluate the effectiveness of a probiotic supplement containing *L. acidophilus* and *B. adolescentis*. The live weight of the experimental ducklings increased as a result of the supplementation. At the end of the 7-week period, the weight of the experimental ducklings was 5.0% higher than that of the control ducklings.

Aliakbarpour *et al*. [14] have reported a positive effect of probiotic supplements containing *B. subtilis* for raising broiler chickens. Similarly, the probiotic Propoul, which contains *L. fermentum*, also has a positive effect; its effectiveness (i.e., increase in live weight, bird livability, and feed costs) has been demonstrated when its powder form added to drinking water is administered daily to male broiler ducks [15]. Moreover, when a 0.05% probiotic supplement (Livesac, Zeus Biotech Limited, India) was used to raise Peking ducks to 8 weeks of age, their live weight significantly increased (p<0.01) [16].

The essential effects of probiotic additives are an increase in the digestibility of feed components and an improvement in feed conversion. Our results show that the average daily feed consumption per head did not differ over 6 weeks, amounting to 157.5 g/head in the control group and 158.5 g/head in the experimental group. Nonetheless, in the experimental ducklings, the feed conversion was 4.7% higher than in the control ducklings. Similar results were obtained after feeding ducks with the probiotic *B. amyloliquefaciens* [11]. The introduction of a probiotic containing lactobacilli and bifidobacteria stimulated protein metabolism. As a result, the total protein and albumin levels in the blood serum of ducklings receiving additional feed increased at 42 days of age. Another study [12] also demonstrated that feeding probiotics containing *Saccharomyces cerevisiae* to broiler ducklings resulted in a significant increase (p<0.05) in protein and albumin levels in the serum.

The average inorganic P content in the serum of the experimental ducklings tended to increase by 4.8% and 1.6% at 21 and 42 days of age, respectively, compared with that in the control group. Moreover, the serum Ca content dynamics differed over time: at 21 days, it was 23.8% higher in the control group than in the experimental group, but at 42 days, it was 9.6% lower in the experimental group. The results of a study by Khaksar *et al*. [17] revealed an increase in the serum Ca concentration when probiotic Aspergillus was used to raise partridges; however, the probiotic did not affect body weight gain.

One of the critical factors that ensure the formation of an effective immune defense is the state and activity of intestinal bacteria. The probiotic supplement in this study contained live lactobacilli and bifidobacteria.

Study conducted on geese fed with *B. subtilis* var. *natto* N21 and *S. cerevisiae* Y10 revealed a considerable increase in the *Lactobacillus* population and a decrease in the *E. coli* population in the small and large intestines [18]. This study showed that the addition of the probiotic supplement allowed the ducklings to digest and assimilate the feed components more efficiently. Thus, the experimental ducklings had a 1.6% higher protein and fat digestibility, 3.4% higher fiber digestibility, and 4.7% higher nitrogen-free extraction fraction digestibility. This indicates the potential for more efficient feed use by poultry receiving this probiotic supplement.

In broiler chickens, *B. subtilis* GM5 was shown to improve feed consumption and digestibility, which is important for reducing production costs [19]. The probiotic supplement affected the meat quality of the experimental poultry. The mass and yield of half-gutted carcasses and the mass of gutted carcasses increased. The muscle output of the experimental birds was 3.5% higher, but their carcass fat content was 1.3% lower than that of the control birds. Other studies [18,20] conducted on broiler ducklings showed that the use of the probiotic strain *L. fermentum* CCM 7158 and *Enterococcus faecium* did not affect the increase in live weight. Nevertheless, it reduced the abdominal fat content [20]. There was also a decrease in lipid concentration and fat deposition in the bodies of the ducklings after probiotic supplementation [21].

## Conclusion

The addition of a probiotic supplement had the most significant impact on microbiocenosis, feed consumption per 1 kg of live weight gain at 4 weeks of age, and meat quality in Pekin ducks, all of which led to increases in the profit associated with farming. As such, raising ducklings using a probiotic supplement containing lactobacilli and bifidobacteria was economically feasible; it provided a 719.3 ruble (9.6 USD) increase in revenue and allowed for an additional profit of 20.6%. Thus, a probiotic supplement containing lactobacilli and bifidobacteria has excellent potential to improve poultry production and is environment-friendly.

## Authors’ Contributions

AK: Analyzed the data. AK, RA, GT, AA, and AB: Drafted the manuscript. AK, RA, GT, AA, and AB: Reviewed and edited the manuscript. RA, GT, and AA: Contributed to conceptualization. AB: Analyzed the samples. AK and RA: Supervised the research. All authors read and approved the final manuscript.
